# Evaluation of Daily Surveillance Blood Cultures During Continuous Renal Replacement Therapy in a Diverse Immunocompromised Population

**DOI:** 10.1017/ash.2025.349

**Published:** 2025-09-24

**Authors:** Anahita Mostaghim, Morgan Walker, Robert L. Danner, Sadia Sarzynski, Alison Han

**Affiliations:** 1 Atrium Health; 2 Critical Care Medicine Department, NIH Clinical Center; 3 National Institutes of Health

## Abstract

**Background:** Immunocompromised patients in the ICU are at high risk of infection. Continuous renal replacement therapy (CRRT) masks fevers. At an institution where one blood culture is routinely obtained daily during CRRT, we evaluated the incidence of positive blood cultures during CRRT. **Methods:** All patients admitted to the NIH Clinical Center receiving CRRT from September 2016 to March 2023 were identified. Charts were abstracted for baseline covariates, laboratory values, microbiology, CRRT days, antimicrobial administration, and mortality. **Results:** A total of 111 patients received CRRT. Ninety-seven (87.4%) had at least one blood culture drawn. Mean age was 43.3 ± 15.8 years and 39 (35.1%) were female. Seventy-four (66.7%) had an underlying malignancy, 36 (32.4%) were neutropenic on CRRT initiation, 32 (28.8%) were post-hematopoietic cell transplant and 9 (8.1%) were post-CAR-T cell therapy. Median CRRT duration was 7 days (IQR 3-16.5). There were 41 separate positive blood culture events, each possibly representing a blood stream infection (BSI), in 27 (24.3%) patients. The most common organism was coagulase-negative Staphylococcus (CoNS) (n=14) followed by Enterococcus faecium (n=8), Candida spp (n=6), and Pseudomonas aeruginosa (n=5). Of 11 cases only growing CoNS, 5 (45.5%) had repeat same-day cultures, but only two grew the same organism. Median time to first positive culture was 13 days (IQR 8-18.5). Fourteen cases (34.1%) were not on matched empiric antimicrobial therapy, of which 4 (28.6%) grew only CoNS. The average number of blood cultures per CRRT day was 1.2. Total number of CRRT days per possible BSI was 34 days, with 98 days for one possible BSI not on matched empiric therapy, and 138 days for a non-CoNS BSI not on matched empiric therapy. Forty-nine (44.1%) patients survived their ICU stay. Of these, 33 (67.3%) continued to have surveillance cultures drawn after CRRT cessation with 16 (32.7%) continuing after ICU discharge. Median days of surveillance cultures after CRRT cessation was 7 days (IQR 5-10). **Conclusion:** While the total proportion of positive cultures not on matched empiric therapy was high at 34.1%, the total number of CRRT days for one non-covered positive culture was high at 98 days. These numbers go down to 24.4% and up to 138, respectively, if CoNS-only cultures are excluded. Routine daily blood cultures may detect a small number of unexpected BSIs in patients whose fever response is masked while on CRRT. However, it is a low yield practice that could benefit from a more targeted approach.

